# 

**Published:** 1892-05

**Authors:** 


### Notice of Dental Meetings.

### Dental Society of the State of New York.

The Dental Society of the State of New York will hold its twenty-fourth annual meeting at Albany, Wednesday and Thursday, May 11th and 12th, 1892.

Papers will be read by the following distinguished members of our profession: Edwin T. Darby, D.D.S., Philadelphia, Pa.; Eugene S. Talbot, D.D.S., Chicago, Ill.; C. F. W. Bodecker, D.D.S., New York City, N. Y.; Albert Carter Westlake, D.D.S., Elizabeth, New Jersey; and discussed by prominent dentists from all parts of the United States. In order that the discussions may be interesting, all those who have been invited to open discussions, will be allowed fifteen minutes—others ten.

Nothing will be left undone to promote the interests of practical and scientific dentistry. No clinics! No exhibits!

C. S. Butler, Secretary.

### Florida State Dental Association.

The annual meeting of this body will be held in Jacksonville, on the 10th day of May, 1892. The State Board of Dental Examiners meet at the same time and place, and also the State Committee of the World's Columbian Dental Congress. This will be an important and interesting occasion for the profession of Florida, and for such other members of the profession as can make it convenient to be present; there will be a large display of instruments, appliances and material. Railroads will make special rates. Hotel rates have been made for $2.00 per day for all attending the Society.

A cordial invitation is extended to all to be present.

### Dental Meeting.

The American Dental Society of Europe will hold its eighteenth annual meeting at Basle, Switzerland, August 1st, 2nd and 3rd.

Members of the profession are cordially invited to attend. Clinics will be a special feature of this meeting. The University will place desirable rooms at the disposal of the Society, and an ingenious amphitheater for accommodating in the immediate vicinity of the patient a larger number of spectators than are able to witness operations under the ordinary circumstances, will be loaned by the Swiss Dental Association. Programmes may be had on application to the President, Dr. Bryan, of Basle, or to

CHAS. W. JENKINS, Sec'y, Dresden.

### Illinois State Dental Society.

The twenty-eighth annual meeting of the Illinois State Dental Society will be held at Springfield, Ill., May 10-13, 1892. The State Board of Dental Examiners meet at the same time and place. The profession generally is cordially invited.

LOUIS OTTOFY, Secretary,
70 Dearborn St., Chicago.

EDITORIAL.

The Ohio Amended Law—Amended ... 250
A Remarkable Man ... 251
The Isaac Knapp Dental Coterie ... 252
Obituary—Dr. John Allen ... 253

### THE DENTAL REGISTER,

**A MONTHLY MAGAZINE DEVOTED TO THE INTERESTS OF THE**

### DENTAL PROFESSION.

*Terms Reduced to* $2.00 *Per Year. Single Copies,* 25 *Cents.*

**EDITED BY PROF. J. TAFT, D.D.S.**

—AND—

**Published by SAM'L A. CROCKER & CO., Ohio Dental and Surgical Depot,**

The volume commences in January and closes in December.

The REGISTER being issued monthly is always fresh, therefore Indispensable to the practitioner who desires to keep posted up with the new inventions and improvements which are daily developed. Neither expense nor effort will be spared to give value and variety to its contents. Articles will be illustrated with well executed engravings whenever they will add to the interest or assist to explanation.

Its extensive circulation and frequency of issue make it one of the BEST DENTAL ADVERTISING MEDIUMS in the United States.

All subscriptions must begin with January or July.

Local or special notices, five lines or less, will be inserted for $3 each insertion; over five lines, 50 cts. per line.

All advertising bills payable in advance, or at furthest, after the issue of the first number containing the advertisement. All transient advertisements to be paid for in advance.

☞CONTRIBUTIONS SOLICITED.

Exclusively Editorial Communications should be addressed to the Editor.

The latest number of the REGISTER may be ordered with or without other goods at any time, and all letters should be addressed to

**SAM'L A. CROCKER & CO., Ohio Dental and Surgical Depot, Cincinnati, O.**

